# Survival rates of adult patients with Hodgkin lymphoma who underwent ABVD versus escalated BEACOPP in a resource‐limited country: An observational study

**DOI:** 10.1002/cnr2.1839

**Published:** 2023-05-31

**Authors:** Tawatchai Suwanban, Supat Chamnanchanunt, Pravinwan Thungthong, Chajchawan Nakhahes, Kunapa Iam‐arunthai, Tananchai Akrawikrai, Udomsak Bunworasate

**Affiliations:** ^1^ Division of Hematology, Department of Medicine, Rajavithi Hospital College of Medicine, Rangsit University Bangkok Thailand; ^2^ Department of Clinical Tropical Medicine, Faculty of Tropical Medicine Mahidol University Bangkok Thailand; ^3^ Division of Hematology, Department of Medicine, Faculty of Medicine Chulalongkorn University Bangkok Thailand; ^4^ Research Unit in Translational Hematology, Faculty of Medicine Chulalongkorn University Bangkok Thailand

**Keywords:** adult, advance‐stage cancer, chemotherapy, Hodgkin disease, life expectancy, limited resource country

## Abstract

**Background:**

The survival rate of adult patients with Hodgkin lymphoma (HL) depends on the responses to standard chemotherapy, radiotherapy, or combined therapy. Resource‐limited countries face numerous obstacles in supporting patients with HL who undergo chemotherapy, especially in advanced stages.

**Aim:**

To analyze the survival outcomes of adult patients with HL after combined‐modality treatment (CMT) with involved‐field or non‐involved‐field radiotherapy.

**Methods and Results:**

We retrospectively reviewed the medical records of 90 adult patients with HL who received CMT at Rajavithi Hospital, Bangkok between 2007 and 2021. Patients with stage I‐IV disease received different therapies depending on their risk group. The risk groups were evaluated according to initial response, bulky disease, and B symptoms. Patients (n = 90) who underwent CMT were followed up for 34.7 months (range, 1–141 months). The median follow‐up periods of early and advanced‐stage patients were 53.1 months and 23.5 months, respectively. The estimated 5‐year overall survival (OS) and progression‐free survival (PFS) rates of patients with advanced‐stage diseases were 85% and 62%, respectively. There was a difference in the 3‐year overall survival among advance‐stage patients who underwent ABVD (94%) compared to those administered BEACOPPesc (50%), and the 3‐year PFS (84%) among patients who underwent ABVD was higher than that among those administered BEACOPPesc (66%). Radiotherapy increased toxicity but did not improve the survival rate.

**Conclusion:**

Chemotherapy administered to patients with advanced‐stage adult HL was more effective than BEACOPPesc when ABVD was administered. Our findings are relevant for hospitals with limited resources.

## 1 INTRODUCTION

Adult Hodgkin lymphoma (adult HL) is an uncommon hematological malignancy with an estimated incidence of 3.5 per 100 000 people.^1^, ^2^, ^3^ In Southeast Asia, adult HL represents approximately 5% of adult lymphoma.^4^, ^5^ The clinicopathological findings of adult HL in Southeast Asia differs from those of patients residing in Western countries.^6^ The survival rate of adult HL patients depends on treatment responses to standard chemotherapy, radiotherapy, or combined therapy. Achieving a longer overall survival (OS) must balance the disease–response to treatment and treatment‐associated toxicity. Patients with early‐stage adult HL experience higher OS rates than those with advanced‐stage HL. Bulky disease adversely influences disease–response–treatment.^7^ The ideal first‐line treatment for advance‐stage adult HL is controversial because of response rates and clinical toxicities.^4^, ^8^, ^9^ For example, there is debate concerning the ABVD (doxorubicin, bleomycin, vinblastine, and dacarbazine) and escalated BEACOPP (BEACOPPesc; escalated bleomycin, etoposide, doxorubicin, cyclophosphamide, vincristine, dacarbazine, and dexamethasone) regimens with respect to their tumor control activities and clinical toxicities.^8^ Five clinical trials of patients residing in Western countries, which compared the ABVD and BEACOPP‐base regimens, found that the relapse rate of the BEACOPP group was lower, although the survival rates between the groups were not significantly different.^8^, ^9^, ^10^, ^11^, ^12^ Unfortunately, resource‐limited countries face numerous obstacles to the support of patients with adult HL who undergo chemotherapy.^2^ Moreover, we are unaware of any report on the long‐term survival or treatment‐related clinical toxicities of patients with early‐ and advance‐stage adult HL at a tertiary hospital with limited resources. Interim evaluation is employed to optimize personalized chemotherapy and radiation therapy (RT).^3^, ^13^ However, diagnostic tools are unavailable in many countries, particularly in resource‐limited countries or countries most affected by the COVID‐19 pandemic.^14^ Therefore, clinical evaluations are required to investigate the outcomes in real‐world settings. Few studies have reported the clinical evaluation of HL, although most have employed new evaluation techniques such as positron emission tomography (PET) combined with computed tomography (PET‐CT).^3^, ^15^, ^16^ Furthermore, most patients were only evaluated for their clinical response to combined‐modality treatment (CMT). Therefore, we determined the clinical survival, disease‐free survival, and toxicities in adult patients with HL who underwent CMT at a tertiary hospital.

## 2 MATERIALS AND METHODS

We reviewed the medical records of patients with HL, aged at least 18 years, between 2007 and 2022. All patients were pathologically diagnosed using a surgical lymph node or percutaneous lymph node biopsy. We enrolled patient (*n* = 90) with tissue pathologies consistent with HL, and excluded those with unavailable medical records, and those who did not undergo CMT. This retrospective and prospective observational study was approved by the Ethical Committee of Rajavithi Hospital (IRB No.159/2564).

**FIGURE 1:**
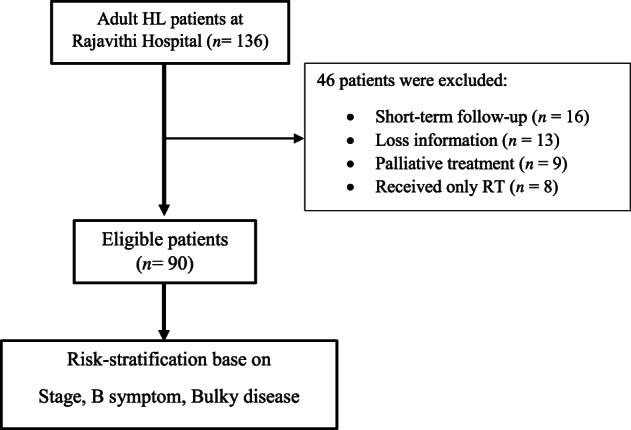
Flow chart with selection criteria and risk‐stratification. Adult HL patients, adult Hodgkin lymphoma; CMT, combined modality treatment.

Patients were examined according to the hospital protocol, which included bone marrow aspiration/biopsy, and computed tomography (CT) of the neck, chest, and whole abdomen. Patients with sufficient economic resource underwent PET‐CT. Patients were classified according to the Ann Arbor Staging (AAS) system which defines a bulky mass as that of a lymph node >10 cm in diameter or a mediastinal mass size > one‐third of the maximal thoracic diameter.^4^ B symptoms included night sweats, noninfectious fever (body temperature ≥ 38°C), and weight loss ≥10% within 6 months. Risk classification was defined according to the initial disease stage, and adverse clinical factors (e.g., presence of bulky disease or ESR ≥50 or B symptoms).^4^ Groups were defined as follows: favorable disease (low‐risk) was AAS I‐II without adverse factors, unfavorable disease (intermediate‐risk) was AAS I‐II with adverse factors, and AAS III‐IV as a high‐risk group. The selection criteria and risk‐stratification are presented in Figure 1. All the patients underwent CMT depending on their risk classification. Most patients received CMT with or without RT according to the NCCN clinical practice guidelines in oncology.^4^ The CMT protocols included ABVD, and BEACOPPesc as base regimens, which were applied base on adult HL studies.^4^, ^5^, ^8^, ^12^, ^17^, ^18^ Disease–response–treatment was assessed after completion of first‐line chemotherapy, as a primary response, or after the administration of complete cycles of each protocol. Adult patients with HL underwent RT within 1 month after the completion of chemotherapy. Target volumes were calculated according to the guidelines.^4^, ^19^, ^20^ The target volume was grossly assessed according to the regions of visibly involved lymph nodes (LNs) in pre‐ and post‐CMT imagines. The RT techniques included involved‐field RT (IFRT) and involved‐nodal RT (INRT). The IFRT protocol mainly involved LNs observed during the pretreatment evaluation, and INRT was defined according to the initial volume of the LNs or that of the residual involved LNs according to diagnostic imaging to contour the clinically‐targeted volume.

After the completion of CMT, clinical evaluation was conducted by two independent physicians who identified LN on diagnostic imaging. Complete remission (CR) was defined a decrease in the mass/LNs size >70% on CT or ultrasonography,^21^, ^22^ absence of the disease as determined by gallium scans based on gallium uptake at tumor site,^23^ or Deauville Criteria score ranging from 1 to 3 for PET‐CT.^4^ A partial response (PR) characterized using CT, was indicated by 50–70% smaller LNs and Deauville Criteria 4 without new LNs according to PET‐CT findings. A stable response (SR) or progressive response was defined as similar or increasing LNs pre‐ and post‐CMT. In these groups, the patients continuously underwent additional CMT. Regular clinical evaluations were performed every 6 months until 2 years after the last CMT, and every year thereafter until loss to follow‐up or death.^4^, ^20^ Toxicities were defined as acute‐ and last‐stages which were evaluated between pre‐and post‐CMT according to available laboratory tests, imaging, pulmonary function tests, and echocardiography. Acute toxicities, which were evaluated during CMT and 1 month thereafter, were classified as chemotherapy‐ and RT‐related toxicities. Patients who underwent the anthracycline based regimen were evaluated using echocardiography at least once after the completion of CMT. The Common Terminology Criteria were used to classify the severity of toxicities.^24^

Data were collected from electronic medical records, and all parameters were analyzed. OS was calculated from the first diagnosis to any cause of death and was censored if patients were lost to follow‐up or information for >12 months. Progression free survival (PFS) was calculated as the time form the first diagnosis to disease progression, relapse, or death. We used the Kaplan–Meier method and log‐rank test to evaluate the significance of the differences in OS rates and PFS between the groups. Hazard ratios (HRs) with 95% confidence intervals (CI) associated with 5‐years survival were calculated using Cox regression models. Statistical analyses were performed using SPSS software for Windows (version 18; IBM: NY; USA). Statistical significance is defined by *p*‐value ≤.05.

## 3 RESULTS

### 3.1 Patients' characteristics

We analyzed the records of 90 patients with HL treated with CMT (Table 1). The median age was 28.5 years (interquartile range [IQR], 24.0–43.5 years) and 49 were women. Most patients presented with lymphadenopathy (*n* = 23; 25.6%). Histopathological findings were as follows; mixed cellularity (*n* = 37), nodular sclerosis (*n* = 32), unclassifiable (*n* = 14), lymphocyte rich (*n* = 6), and lymphocyte depletion (*n* = 1). The other presenting symptoms were B symptoms (*n* = 19; 21.2%), weight loss (n = 10; 11.1%), bulky disease (*n* = 21; 23.3%), extranodal involvement (*n* = 8; 8.9%), and spleen involvement (*n* = 3; 3.4%). Eight patients presented with extranodal involvement (three with splenic involvement). The most frequent finding was AAS II, followed by AAS III (*n* = 24; 26.7%), AAS IV (*n* = 18; 20%), and AAS I (*n* = 8; 8.9%). Thirty‐three (36.7%), 15 (16.7%), and 42 (46.7%) patients were classified as having low‐, intermediate‐, and high‐risk HL, respectively, and 10 patients (11.1%) presented with a comorbidities. All the patients underwent CMT according to the stage and risk‐stratification protocol.

**TABLE 1:** Baseline characteristic of adult Hodgkin lymphoma (*n* = 90).

Characteristic	Value	
Age (year)	28.5 (24.0–43.5)
Sex	
Male	41 (45.6)
Female	49 (54.4)
Ann Arbor Staging	
I	8 (8.9)
II	40 (44.4)
III	24 (26.7)
IV	18 (20.0)
Histologic type	
Mixed cellularity	37 (41.1)
Nodular sclerosis	32 (35.6)
Unclassifiable	14 (15.6)
Lymphocyte rich	6 (6.7)
Lymphocyte depletion	1 (1.1)
Lymphadenopathy	23 (25.6)
Presenting symptom	
Weight loss	10 (11.1)
Fever at night time	6 (6.7)
Fatigue	5 (5.6)
Dyspnea	4 (4.4)
Night sweating	3 (3.3)
Itching	1 (1.1)
B symptoms	
Yes	19 (21.2)
No	71 (78.8)
Bulky disease	
Yes	21 (23.3)
No	69 (76.7)
Extranodal involvement	
Yes	8 (8.9)
No	82 (91.1)
ECOG performance status	
0	59 (65.6)
1	27 (30.0)
2	4 (4.4)
Risk classification	
Low risk group	33 (36.7)
Intermediate risk group	15 (16.7)
High risk group	42 (46.7)
Comorbidity	
Present	10 (11.1)
Absent	80 (88.9)

*Note*: Values are showed as median (interquartile range) or number (percentage).

### 3.2 Treatment and response

First‐line chemotherapy included six cycles of ABVD/ABV (*n* = 83; 92.2%) every 2 weeks or 6–8 cycles of BEACOPPesc (*n* = 7; 7.8%) every 4 weeks, according to the physician's discretion. Disease–response to chemotherapy was assessed according to the initial staging method (physical examination and diagnostic imaging). Patients were evaluated using PET‐CT (*n* = 13; 14.4%), CT (*n* = 34; 37.8%), gallium scan (*n* = 14), and ultrasonography (*n* = 29). Among the 63 (70.0%) patients who achieved CR as the primary response, 21 (23.3%) had progressive HL. Thirty‐three patients who achieved CR and 10 HL patients with relapsed disease were evaluated using gallium scan and ultrasonography. After completing chemotherapy, 38 patients received RT (IFRT or INRT). Twenty‐six patients with early stage HL (68.4%) who received radiotherapy showed a significantly higher proportion of advance‐stage HL (31.6%) combined radiation (*p* = .014). The median RT dose was 40.0 Gy (IQR; 31.5–50.0 Gy) (Table 2). Two patients receiving the ABVD/ABV regimen developed complications (cardiac and secondary malignancies). Among the 27 (30%) patients who did not respond to first‐line chemotherapy, 20 (71.4%) continued to receive second‐line chemotherapy (ICE‐based regimen), and 5 patients (38.5%) underwent third‐line chemotherapy (ESHAP regimen).

**TABLE 2:** Summary of combined modality treatment and median follow‐up time according to Hodgkin lymphoma classifications among adult Hodgkin lymphoma patients.

Parameters	Value	*p*‐Value
Chemotherapy		
ABVD	80 (88.9)	
BEACOPPese	7 (7.8)	
ABV	3 (3.3)	
Radiation therapy (RT)		
Yes	38 (42.2)	
No	52 (57.8)	
RT dose (Gy)	40.0 (31.5–50.0)	
Response after completion CMT		
Complete response	63 (70.0)	
Progressive response	21 (23.3)	
Partial response	5 (5.6)	
Stable response	1 (1.1)	
Ann Arbor Staging and median follow‐up time (months)		.02
I	65.7 (33.3–89.3)	
II	46.1 (20.8–71.9)	
III	24.1 (13.7–61.7)	
IV	18.4 (10.6–41.8)	
All patients	34.7 1–141 months	
Stage classification and median follow‐up time (months)		.005
Early stage	53.1 (21.6–73.5)	
Advance stage	23.5 (12.7–46.6)	
Risk classification and median follow‐up time (months)		.01
Low risk group	40.0 (20.0–72.0)	
Intermediate risk group	33.0 (14.5–63.0)	
High risk group	14.0 (7.0–23.0)	

*Note*: Values are showed as median (interquartile range) or number (percentage).

### 3.3 Treatment outcomes

The median follow‐up time for all patients was 34.7 months (range, 1–141 months). Follow‐up periods according to AAS stage were as follows: AAS I, 65.7 months (IQR, 33.3–89.3 months); AAS II, 46.1 months (IQR, 20.8–71.9 months); AAS III, 24.1 months (IQR, 13.7–61.7 months); and AAS IV, 18.4 months (IQR, 10.6–41.8 months) (*p* = .02). The median follow‐up duration differed significantly between patients with AAS stages II and IV (*p* = .007). There was a significant difference (*p* = .005) between the median follow‐up times among patients with early‐stage (53.1 months; IQR, 21.6–73.5 months) and those with advance‐stage disease (23.5 months; IQR, 12.7–46.6 months). Furthermore, there was a significant difference (*p* = .01) in the median follow‐up times between patients at low risk (40.0 months; IQR, 20.0–72.0 months), intermediate risk (33.0 months; IQR, 14.5–63.0 months), or high risk (14.0 months; IQR, 7.0–23.0 months) (Table 2).

**FIGURE 2:**
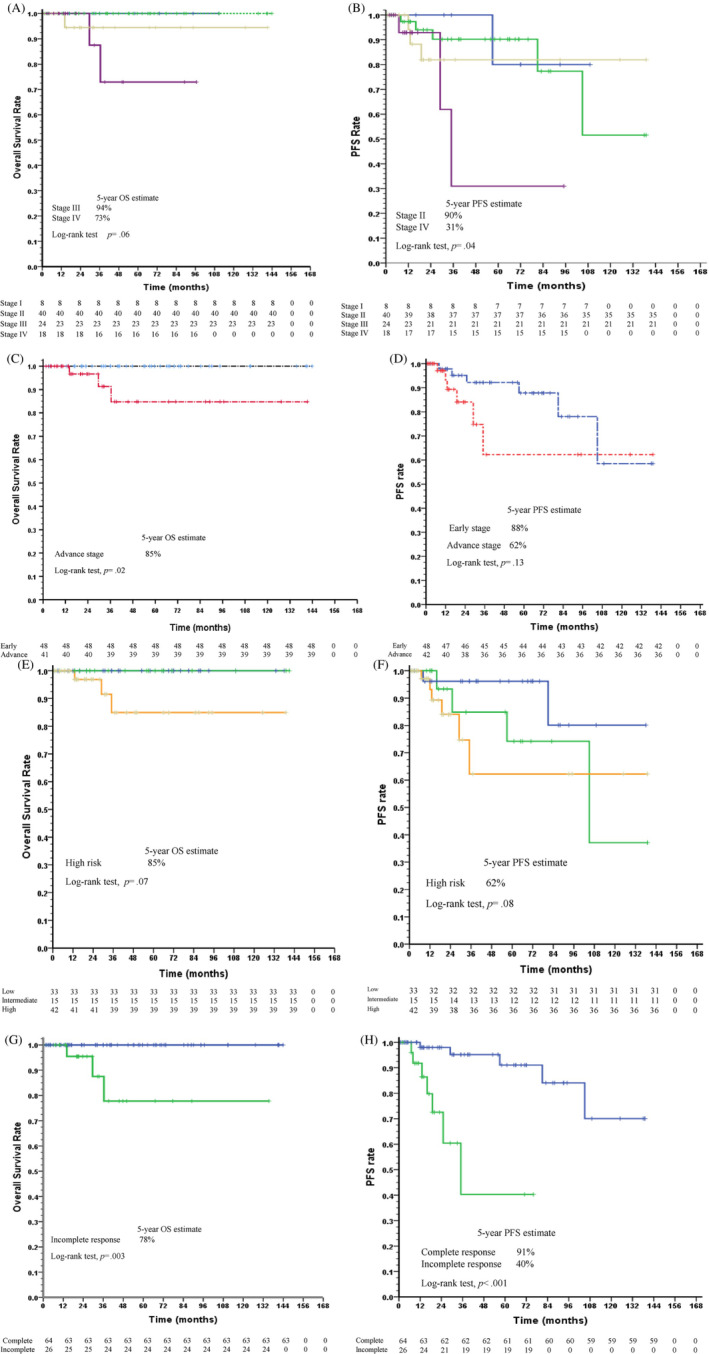
Kaplan–Meier curves for (A) overall survival in adults Hodgkin lymphoma for stage I (blue), stage II (green), stage III (yellow), and stage IV (purple). (B) Progression free survival in adults Hodgkin lymphoma for stage I (blue), stage II (green), stage III (yellow), and stage IV (purple). (C) Overall survival for early stage (blue), advance stage (red). (D) Progression free survival for early stage (blue), advance stage (red). (E) Overall survival for low risk group (blue), intermediate risk group (green), high risk group (yellow). (F) Overall survival stage for low risk group (blue), intermediate risk group (green), high risk group (yellow). (G) Overall survival rate in adult HL patients with complete treatment response (blue), incomplete treatment response (green). (H) Progression free survival for adult HL patients with complete treatment response (blue), incomplete treatment response (green).

The 5‐year OS and PFS rates of all patients with HLs were 95% and 81%, respectively. The 5‐year OS rates of patients with AAS III and IV were 94% and 73%, respectively. The OS did not significantly differ among AAS stages (*p* = .06). In contrast, the 5‐year PFS rates of patients with AAS II (90%) and AAS IV (31%) were significantly different (*p* = .04) (Figure 2A,B). There were significant differences in the 5‐year OS (85%) (*p* = .02), but PFS in the early stage (88%) was not significantly different from that in the advanced (62%) (Figure 2C,D). The estimated 5‐year OS and PFS rates of the high‐risk group were 85% (*p* = .069) and 62% (*p* = .08), respectively (Figure 2E,F). All patients who responded to chemotherapy had significantly longer 5‐year OS and PFS than those with an incomplete response (OS = 78%, *p* = .003; PFS = 91%, *p* < .001) (Figure 2G,H). Patients' records of advance adult HL patients were collected for only 3 years; 3‐year OS rates of those who underwent treatment with ABVD (94%) and BEACOPPesc (50%), and 3‐year PFS rates of those who underwent treatment with ABVD (84%), and BEACOPPesc (66%). There were no significant differences in 5‐year OS and PFS rate between patients with advanced disease who underwent radiotherapy and those who did not (data not shown). Moreover, 3 patients died (one each of pneumonia, secondary lymphoma, and sudden cardiac arrest). Two adult patients with HL treated with CMT developed cardiotoxicity. As shown in Table 3, adult HL patients with a low ECOG score (adjusted HRs = 3.59; 95%CI 1.05, 12.17), early treatment response (adjusted HRs = 10.05; 95% CI 2.38, 42.37) after chemotherapy showed a significantly progression free survival rate (*p* = .04 and .002, respectively).

**TABLE 3:** Cox's proportional hazard model for risk factors associated with progression free survival and overall survival among Hodgkin lymphoma patients.

Factors	Progression free survival				Overall survival			
	Crude HRs (95%CI)	*p*‐Value	Adjusted HRs (95%CI)	*p*‐Value	Crude HRs (95%CI)	*p*‐Value	Adjusted HRs (95%CI)	*p*‐Value
Age (ref = younger age)	1.0 (0.96–1.04)	.91	–	–	1.0 (0.92–1.08)	.98	–	–
Gender (ref = male)	0.52 (0.16–1.62)	.25	–	–	0.01 (0–145.89)	.34	–	–
Stage (ref = advance)	0.42 (0.13–1.35)	.15	–	–	140.91 (0.01–855.99)	.33	–	–
Tissue type (ref = classical type)	0.62 (0.16–2.32)	.48	–	–	0.37 (0.33–4.13)	.42	–	–
Extra lymphadenopathy (ref = present)	0.32 (0.06–1.48)	.15	–	–	0.21 (0.19–2.34)	.21	–	–
B symptom (ref = present)	0.43 (0.13–1.49)	.18	–	–	0.41 (0.36–4.59)	.46	–	–
Bulky disease (ref = present)	1.38 (0.42–4.60)	.59	–	–	31.72 (0–67.07)	.54	–	–
ECOG (ref = Low ECOG)	4.05 (1.21–13.49)	.02	3.59 (1.05–12.17)	.04	0.26 (0.36–44.01)	.26	–	–
Weight loss (ref = present)	0.53 (0.12–2.48)	.43	–	–	0.44 (0–7.41)	.74	–	–
Dyspnea (ref = present)	0.16 (0.017–1.39)	.09	–	–	21.28 (0–9.75)	.82	–	–
Night sweat (ref = present)	0.34 (0.04–2.67)	.30	–	–	21.40 (0–2.24)	.81	–	–
Itching (ref = present)	20.58 (0–6.41)	.84	–	–	20.48 (0–3.04)	.92	–	–
Bone marrow involvement (ref = present)	0.26 (0.05–1.34)	.11	–	–	0.75 (0.01–0.82)	.34	–	–
Treatment response (ref = complete response)	10.75 (2.64–43.76)	.001	10.05 (2.38–42.37)	.002	6.53 (0.58–73.23)	.12	–	–

Abbreviation: ECOG, Eastern Cooperative Oncology Group.

## 4 DISCUSSION

Here, we reported a high incidence of newly diagnosed HL in young adults with a median age of 28.5 years. This observation is similar to that of other studies indicating an increased incidence of HL among young adults.^1^, ^8^, ^15^ Furthermore, our findings, which show that approximately 25% of adult HL patients exhibited B symptoms and experienced a low incidence of bulky disease, are consistent with those of previous studies.^16^, ^25^ Adult HL comprises a high‐rate subtype with mixed cellularity, consistent with the findings of Evens et al.^25^ Our patient characteristics upon diagnosis, according to the Eastern Cooperative Oncology Group criteria are similar to those of prior studies.^8^, ^10^, ^11^, ^12^ However, our present patients were mainly classified with AAS stage II HL. Risk factors for survival include complete response to first‐line CMT (with a hazards ratio of 10 times), which may increase OS and PFS, whereas, a late treatment response for CMT has limited efficacy. The present study's clinical evaluation of criteria associated with the response to CMT is similar to that reported by other advance studies.^26^, ^27^

The median follow‐up of patients with AAS III and IV was 34 months (range, 14–68 months), which is shorter than that of patients with other stages. The PFS and OS rates of patients with stage IV AAS were significantly lower than those of patients with other AAS stages. These findings are similar to those of other studies of patients with AAS IV.^28^ Here, we found that the 5‐year OS differed according to favorable classification (advanced stage), and that risk‐stratification (high‐risk group) revealed significantly shorter rates compared with the low‐intermediate risk groups. Furthermore, our patients received a radiation dose of 40 Gy, equal to that employed by van Nimwegen and colleague.^29^ The early‐stage groups significantly treat chemotherapy with RT than advance‐stage. An advanced stage may comprise numerous metastatic LNs that are incurable using local radiotherapy.^30^ In contrast, this finding is associated with an increased risk of mortality in patients who undergo RT. For example, van Nimwegan et al., (2016) and Schaapveld et al., (2015) found that cardiac toxicity and the risk of secondary tumors increased among such patients.^29^, ^31^

The main unanswered question is: What is the optimal regimen to improve survival among advanced stage adult HL patients? Therefore, we focused our analyses on adult patients with advanced stage HL. To our knowledge, there is a lack of evidence indicating the efficacy of the bleomycin‐based regimen (ABVD) and the intensive regimen (BEACOPPesc) when applied in limited‐resource countries. Our findings revealed differences in the 3‐year OS rates between patients with advanced HL treated with ABVD (94%) and BEACOPPesc (50%). However, inferior 3‐year PFS was experienced by patients in the BEACOPPesc group (66%), which is inconsistent with other studies, including the EORTC 20012 and HD2000 studies.^8^, ^9^, ^12^, ^32^

Our present analysis of data in a resource‐limited country shows that ABVD is more suitable, although there were a small number of patients in the BEACOPPesc group. We noted a slight decrease in 3‐year OS (94%) and PFS (84%) among patients with advanced stage HL who received the ABVD regimen. Notably, we show that the rate of disease progression and interruptions in toxicity in patients with advanced disease were highly associated with BEACOPPesc. For example, such patients experienced lower OS (50%) compared with studies reporting OS ranging from 85% to 97%.^33^, ^34^, ^35^, ^36^

Although few of our patients were evaluated using interim PET‐CT during chemotherapy, they experienced severe acute hematologic toxicities similar to those of other studies.^8^, ^9^, ^10^ Refining HL patients undergoing treatment is crucial with PET‐CT, but our study had limitations. The limitations of our study include its retrospective and prospective design study and the small number of patients with advanced disease who underwent BEACOPPesc therapy. However, this study demonstrated a high survival rate and reduced disease progression among patients with advanced stage HL who received the ABVD regimen. The selection of the chemotherapy protocol may be influenced by aggressive disease, age, and physician concerns; this requires more prospective multicenter trials in the future. Our study's strengths included the relatively long follow‐up of patients with early‐stage and of patients with advanced‐stage cancer who were administered ABVD therapy. Additional prospective studies including patients who received BEACOPP therapy for advanced stage adult HL are required.

In conclusion, our study supports ABVD as a well‐tolerated chemotherapy regimen that is effective in controlling advance‐stage tumors with higher survival rates than BEACOPPesc. These findings highlight its role as a first‐line treatment for advance‐stage adult patients with HL in resource‐limited countries.

## AUTHOR CONTRIBUTIONS

**Tawatchai Suwanban:** Conceptualization (equal); data curation (equal); formal analysis (equal); funding acquisition (equal); investigation (equal); methodology (equal); resources (equal); software (supporting); supervision (supporting); validation (equal); visualization (equal); writing – original draft (equal); writing – review and editing (equal). **Supat Chamnanchanunt:** Conceptualization (equal); data curation (equal); formal analysis (equal); funding acquisition (lead); investigation (equal); methodology (equal); project administration (lead); resources (equal); software (equal); supervision (equal); validation (lead); visualization (equal); writing – original draft (equal); writing – review and editing (lead). **Pravinwan Thungthong:** Data curation (equal); formal analysis (equal); investigation (equal); methodology (equal); supervision (supporting); validation (supporting); writing – original draft (supporting); writing – review and editing (equal). **Chajchawan Nakhahes:** Data curation (equal); investigation (equal); methodology (equal); resources (supporting); supervision (supporting); validation (supporting); writing – review and editing (supporting). **Kunapa Iam‐arunthai:** Data curation (supporting); investigation (equal); methodology (equal); validation (supporting); visualization (supporting); writing – review and editing (supporting). **Tananchai Akrawikrai:** Investigation (supporting); methodology (equal); validation (supporting); visualization (supporting); writing – review and editing (supporting). **Udomsak Bunworasate:** Conceptualization (equal); data curation (equal); formal analysis (equal); project administration (lead); resources (supporting); supervision (equal); validation (equal); visualization (equal); writing – review and editing (equal).

## CONFLICT OF INTEREST STATEMENT

The authors have stated explicitly that there are no conflicts of interest in connection with this article.

## ETHICS STATEMENT

The Ethical Committee of Rajavithi Hospital approved this retrospective and prospective observation study (IRB No.159/2564). Informed consent were performed for prospective observation study.

## Data Availability

Data sharing is not applicable to this article as no new data were created or analyzed in this study.
